# Quality of life of children with achondroplasia and their parents - a German cross-sectional study

**DOI:** 10.1186/s13023-019-1171-9

**Published:** 2019-08-09

**Authors:** Stefanie Witt, Beate Kolb, Janika Bloemeke, Klaus Mohnike, Monika Bullinger, Julia Quitmann

**Affiliations:** 1 Department of Medical Psychology, University Medical Center Hamburg-Eppendorf, Martinistraße 52 | W 26, 20246 Hamburg, Germany; 2 University of Applied Sciences Hamburg, Alexanderstraße 1, 20099 Hamburg, Germany; 3 Institute of Medical Psychology, University of Hamburg-Eppendorf, Martinistraße 52 W26, 20246 Hamburg, Germany; 4 University Hospital Magdeburg, Leipziger Straße 44, Haus 10, 39120 Magdeburg, Germany

**Keywords:** Quality of life, Achondroplasia, Children, Parents, Rare diseases, Special health condition

## Abstract

**Background:**

Achondroplasia is the most common form of disproportionate short stature and might affect not only the quality of life of the affected child but also that of the parents.

**Objectives:**

We aimed to investigate the quality of life of children with achondroplasia from child- and parent perspective as well as the parental quality of life.

**Methods:**

Forty-seven children with achondroplasia and 73 parents from a German patient organization participated. We assessed children’s quality of life using the generic Peds QL 4.0™ as self-reports for children aged 8–14 and parent-reports for children aged 4–14 years. Parental quality of life we assessed using the short-form 8-questionnaire.

**Results:**

Children with achondroplasia showed significantly lower quality of life scores compared to a healthy reference population from both the child- and parent-report (p = ≤.01), except the child-report of the emotional domain (*t* (46) = − 1.73, *p* = .09). Parents reported significantly lower mental health in comparison with a German reference population (*t* (72) = 5.64, *p* ≤ .01) but no lower physical health (*t* (72) = .20, *p* = .85). While the parental quality of life was a significant predictor of parent-reported children’s quality of life (*F* (6,66) = 2.80, *p* = .02), it was not for child-reported children’s quality of life (*F* (6,66) = .92, *p* = .49).

**Conclusions:**

Achondroplasia is chronically debilitating. Thus special efforts are needed to address patients’ and parent’s quality of life needs. This special health condition may influence the daily life of the entire family because they have to adapt to the child’s particular needs. Therefore, clinicians should not only focus on the child’s quality of life but also those of the parents.

## Background

Achondroplasia is the most recognizable form of short stature [1], characterized by disproportionate short stature with prevalence rates about 1:10,000 to 1:30,000 per live births [2, 3]. The cause of achondroplasia was identified to be a gain-of-function mutations in the gene for the fibroblast growth factor receptor 3 (FGFR-3) and is known to be an autosomal dominant trait [1, 4]. Achondroplasia is characterized by short stature, short limbs, and rhizomelic disproportion, macrocephaly, and midfacial retrusion. Other characteristics are a small chest, thoracolumbar kyphosis, lumbar hyperlordosis, limited elbow extension, short fingers, and trident configuration of the hands. Patients may also show hypermobile hips and knees, bowing of the mesial segment of the legs as well as hypotonia [1]. Affected patients experience various orthopedic and neurological complications and might face multiple medical and non-medical challenges in their daily life [5–8].

Adult patients reported physical and mental impairments as well as lower quality of life and lower self-esteem than healthy relatives [9, 10]. By the same time, relatives rated the perception of the condition more frequently as serious or lethal than the affected adults [9]. Furthermore, stigmatization, isolation, and difficulties in accessing adequate healthcare increase the risk for problems in psychosocial health [11, 12].

Therefore, quality of life as a central patient-reported outcome measure became an important health indicator in the context of the care of chronically ill patients [13, 14]. According to this relevance, we have developed and tested a patient-reported outcome instrument for young people with achondroplasia, emphasizing the benefits of this disease-specific instrument [15, 16].

Previous studies reported a significantly reduced quality of life of children with chronic health conditions from both the child and parent perspective in comparison with healthy reference populations [17, 18]. At the same time, other studies reported no differences in quality of life of chronically ill children, compared to healthy peers [19, 20]. In contrast to children with endocrine short-stature, children with achondroplasia reported significantly lower quality of life [8, 12, 19]. In particular, young patients with achondroplasia showed the lowest quality of life scores in the physical domain, the highest quality of life scores in the emotional domain [6, 8, 10, 12, 21].

Results from other studies reveal that young patients rate their quality of life significantly higher than their parents, although the age of the patients can affect the perceived quality of life [8, 12, 21]. The fact that a positive attitude towards body height and weight is significantly associated with a better psychological status has been reported in studies with clinical populations [8, 12, 19]. Besides, results reveal interventions can have a positive effect on the quality of life of young patients with achondroplasia [12, 19].

The impact of chronic illness on both the children themselves and their families has been recognized as an essential research area [22–24]. While the child’s health condition can affect the parental quality of life negatively, competent parenting is regarded to have the potential to positively affect children’s adjustment to their chronic illness [25].

Currently, research on the quality of life of parents of chronically ill children shows no consistent results. While the majority of studies reported limitations in quality of life of parents of chronically ill children [26–29], other studies have shown no significant association between a chronic health condition of a child and parental quality of life [30–33]. We have already studied the quality of life of children, adolescents and young adults with achondroplasia in previous studies [8, 19, 21, 34] in order to [1] understand the patients’ perception of their quality of life, [2] identify psychosocial and clinical predictors for intervention planning and [3] evaluate the effect of a novel intervention [8, 12, 19, 21]. The qualitative analyses showed, that parents – especially when receiving the diagnosis – feel helpless and overwhelmed independently from prenatal and postnatal diagnoses [21]. Gollust, Thompson [35] showed that adults with higher quality of life were more likely to decline prenatal screening than those with lower quality of life. However, we did not yet consider the quality of life of parents with children with achondroplasia.

In the current study, we aimed [1] to investigate the effects of achondroplasia on the quality of life of children with achondroplasia from child and parent perspective, [2] to investigate the quality of life of the parents and [3] to explore associations between parental quality of life and children’s quality of life. We hypothesize that the quality of life of children born with achondroplasia and the parental quality of life will be significantly reduced compared to a reference population. We assume height will be an important variable to explain both, the self and parent-reported children’s quality of life, while parental quality of life will primarily contribute to explain parent reported children’s quality of life.

## Methods

**Fig. 1:**
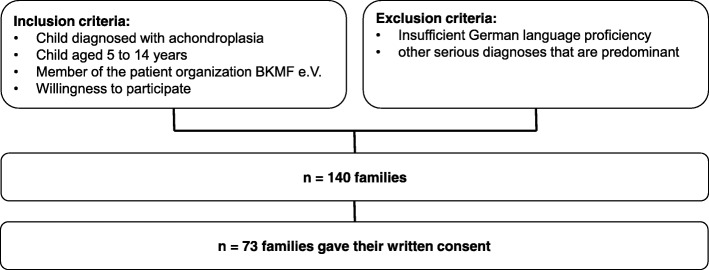
Flowchart of participating families as well as inclusion and exclusion criteria

The present study is part of the Achondroplasia Personal Life Scale Experience Scale study that focused on developing a questionnaire to assess quality of life and functioning in children and adolescents with achondroplasia based on the International Classification of Functioning, Disability, and Health [15]. The current analyses used data from the field test phase. We enrolled families (one parent per child) through the German Association for People of Short Stature and their Families if they met the inclusion and exclusion criteria (Fig. 1). The German Association for People of Short Stature and their Families is a national patient organization focusing on all kinds of short stature and offering many support options such as contact to specialized physicians, orthopedists, psychologists as well as frequent membership meetings to come together with other families with short-statured children. The patient organization contacted only families with a clinical diagnosis of achondroplasia and no other known serious illnesses. Families received detailed information about the aims of this study in a written form. We obtained ethic approval to conduct the study from the Ethical Review Board of Magdeburg, Germany (45/15) before we started.

We have sent out questionnaires for children aged 8 to 14 years and parents of children aged 5 to 14 years in October 2015 by mail to all families that had given their written consent. Data collection took place over six months in which a maximum of two reminders was sent out.

### Measurements

We measured *children’s QoL* using the generic PedsQL 4.0™ questionnaire. This instrument is available in self-report for children aged eight years and over and in parent-report for younger children. The PedsQL 4.0™ is composed of 23 items rated on a 5-point Likert scale and comprises four domains (Physical Functioning, Emotional Functioning, Social Functioning, and School Functioning) as well as three overall scores (Psychosocial Health Summary Score, Physical Health Summary Score, and Total Score). Good reliability was found in the current sample, with Cronbach’s Alpha values ranging from α = .75 (school; parent-report) to α = .94 (total score; self-report). References from a healthy pediatric sample are available for self- and parent-reports [17, 36].

We measured *parental quality of life* using the Short-Form-8 questionnaire (SF-8), which is a short form of the SF-36, an internationally used generic quality of life instrument. In the current sample, the total scores of the SF-8 showed good reliability for the physical component score with Cronbach’s α = .86 and the mental component score with Cronbach’s α = .85. Gender- and age-adjusted German norm-based data are available [37].

Finally, we collected sociodemographic and clinical data from the parents. The standard deviation score (SDS) of height was calculated using German reference values [38].

### Statistics

We performed bivariate analyses using Students t-Test. To determine the degree of convergence between parental quality of life and children’s quality of life, we calculated paired Pearson correlations. We performed multiple linear regression analyses with children’s quality of life (self-report and parent-report) as an endpoint.

For all statistical analyses, we used the *IBM Statistical Package for Social Sciences Statistics* version 21.0. Except for sociodemographic and clinical variables, we replaced missing values by the individual mean score for each variable, if missing data were random and less than 20% of the values. The significance level was set to be less than .05 (*p* ≤ .05).

## Results

A total of 73 families participated, including 73 parent-reports (children aged 5–14 years) and 47 child-reports (children aged 8–14 years) (Table 1).

**Table 1:** Sample characteristics of the participating families

Child age (years) (*n* = 73)	Mean/Median	9.75/9.59
	SD/SE	3.02/.35
Child gender (*n* = 73)	Male	37 (50.7%)
	Female	36 (49.3%)
SDS of height (*n* = 72)	Mean / Median	−5.25 / -5.24
	SD / SE	1.26 / .15
Body mass index (z-scores) (*n* = 71)	Mean / Median	1.36 / 1.41
	SD / SE	.83 / .10
Proportionality ratio (*n* = 68)	Mean / Median	1.82 / 1.86
	SD / SE	.30 / .04
Additional complications ^a^ (*n* = 66–73)	Reduced foramen magnum size	23 (31.5%)
	Hydrocephalus	3 (4.1%)
	Spinal canal narrow	8 (11.1%)
	Tibial bowing or leg bowing	34 (51.5%)
	Chronic middle ear infections	45 (61.6%)
	Tonsillectomy	45 (61.6%)
	Sleep apnea.	30 (41.7%)
	Nightly snoring	64 (88.9%)
	Mouthbreathing	58 (79.5%)
	Daily pain	21 (28.8%)
	Daily fatigue	22 (30.1%)
Height of the children’s mothers in cm (*n* = 71)	Mean / Median	178,13 / 168,00
	SD / SE	99,23 / 11,78
Height of the children’s fathers in cm (*n* = 68)	Mean / Median	188,87 / 178,00
	SD / SE	100,30 / 12,16
Parental gender (*n* = 73)	Male	17 (23.3%)
	Female	56 (76.7%)

^a^ multiple complications possible.

Abbreviations: standard deviation (SD), standard error (SE)

### Children’s quality of life

In the sample, parents of children with achondroplasia reported significantly lower quality of life scores for their children compared to a healthy reference population for all domains (*p* ≤ .01) [17]. The children themselves also rated their quality of life significantly lower compared to a healthy reference population for all domains (*p* ≤ .02) with the exception of the domain emotional (*t* (46) = − 1.73, *p* = .09) (Table 2).

**Table 2:** Descriptive analyses of quality of life dimensions and comparison with norm values

Perspective	Domains	*N*	Mean	SD	Reference-values	t	df	*p*-value
Child-reported quality of life of children with achondroplasia (PedsQL)	Physical	47	74.54	21.23	87.53	−4.14	46	≤.01**
	Emotional	47	73.51	23.03	79.33	−1.73	46	.09
	Social	47	73.40	20.57	85.15	−3.92	46	≤.01**
	School	47	73.59	20.64	81.12	−2.50	46	.02*
	Psychosocial	47	73.51	18.34	81.87	−3.13	46	≤.01**
	Total	47	73.76	18.04	83.84	−3.83	46	≤.01**
Parent-reported quality of life children with achondroplasia (PedsQL)	Physical	72	59.58	23.48	84.48	−9.06	72	≤.01**
	Emotional	73	65.93	19.24	81.31	−6.83	72	≤.01**
	Social	73	61.64	19.04	83.70	−9.90	72	≤.01**
	School	72	67.67	17.52	78.83	−5.44	72	≤.01**
	Psychosocial	73	65.08	15.15	81.65	−9.35	72	≤.01**
	Total	73	63.70	15.83	82.70	−10.25	72	≤.01**
Parental self-reported quality of life (SF-8)	Physical component score	73	50.50	8.49	50.30	.20	72	.85
	Mental component score	73	46.51	10.22	53.25	−5.64	72	≤.01**

Abbreviations: standard deviation (SD), *p < .05, **p < .01

### Parental quality of life

We compared the quality of life scores of the parents of this study sample with norm values of a German reference population and found significantly lower scores for the mental component score (*t* (72) = 5.64, p ≤ .01), but we detected no differences for the physical component score (*t* (72) = .20, *p* = .85) Table 2).

### Correlations between children’s quality of life and parental quality of life

**Fig. 2:**
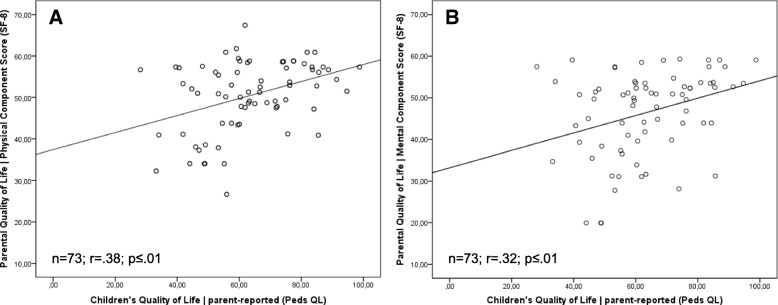
**a**/**b** Pearson correlations between parental quality of life and parent-reported children’s quality of life

**Fig. 3:**
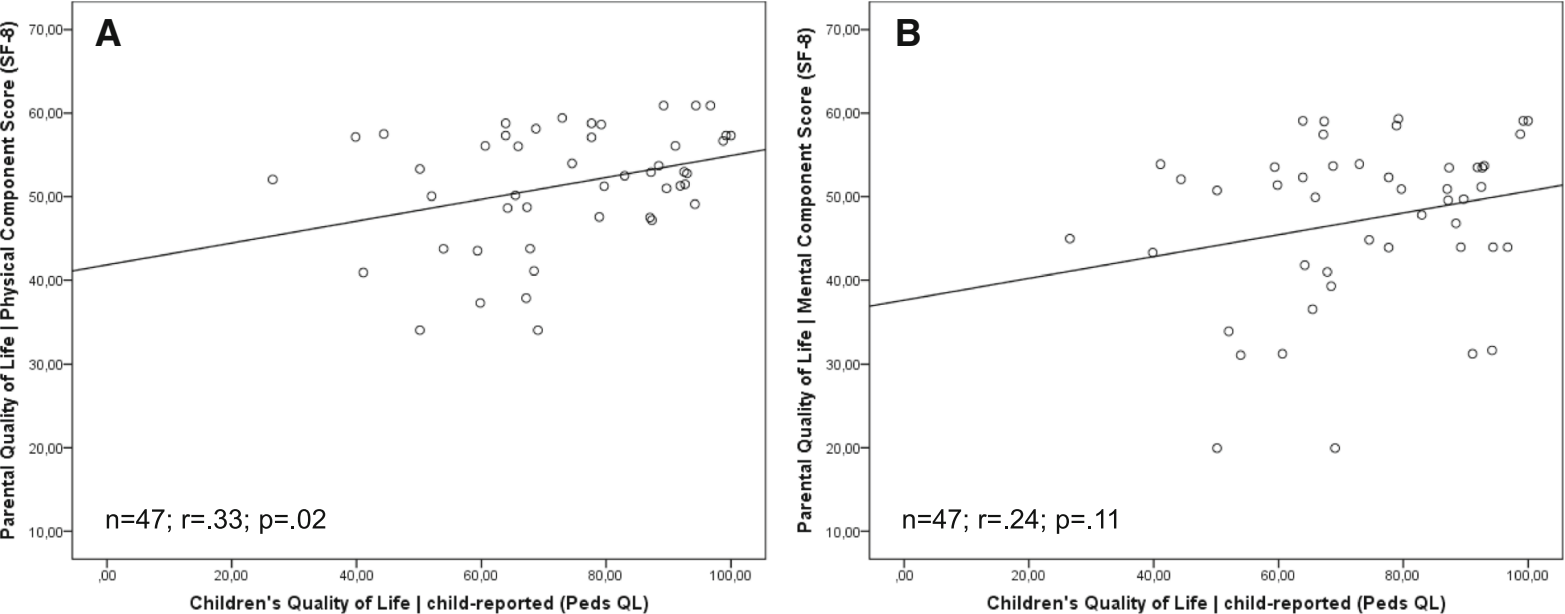
**a**/**b** Pearson correlations between parental quality of life and child-reported children’s quality of life

We show paired Pearson correlations between parental quality of life (physical component score and mental component score), children’s self-reported quality of life and children’s parent-reported quality of life in (Figs. 2 and 3). The values indicate positive correlations with the relatively highest convergence between children’s parent-reported generic quality of life and parental physical quality of life and (r = .38; *p* ≤ .01) as well as children’s parent-reported quality of life and parental mental quality of life (r = .32; *p* ≤ .01). Correlation between child-reported children’s quality of life and parental physical quality of life also reached the significance level (r = .33; *p* = .02). Child-reported children’s quality of life and parental mental quality of life did not show any significant correlation (r = .24; *p* = .11).

With the regression analyses, we demonstrated that our model predicts significantly on the parent-reported children’s quality of life (*F* (6,66) = 2.80, *p* = .02). Parental physical quality of life (*β* = .25, *p* = .04) and parental mental quality of life (*β* = .30, *p* = .02) showed to be significant predictors of parent-reported children’s quality of life. The same model did not contribute to explaining a substantial variance of child-reported children’s quality of life (*F* (6,66) = .92, *p* = .49). For sociodemographic and clinical variables, we did not find any effect on children’s quality of life, neither for the child-reported nor for the parent-reported children’s quality of life (Table 3).

**Table 3:** Multivariate linear regression: Predictors of children’s quality of life from both child-report and parent-report

Independent variables	Children’s quality of life			
	Child-reported		Parent-reported	
	β	*t*	β	*t*
Child’s age	−.02	−.14	−.03	−.23
Child’s gender ^a^	.04	.27	.05	.13
SDS of height	.06	.39	−.04	−.27
Body mass index (z-scores)	−.08	−.56	−.16	−1.12
Proportionality ratio	−.12	−.97	−.12	−1.03
Physical component score (SF-8)	.18	1.35	**.34**	2.72**
Mental component score (SF-8)	.15	1.13	**.26**	2.12*
Parental gender ^b^	.01	.07	−.05	−.43
Model summary	**R**^**2**^ **= .09**		**R**^**2**^ **= .23**	
	**F**_**(8,64)**_ **= .82**		**F**_**(8,64)**_ = 2.34*	

^a^child’s gender: 0 – male, 1 – female

^b^ parental gender: 0 – male, 1 – female

* *p* ≤ .05 ** p ≤ .01

## Discussion

To our knowledge, this is the first study focusing on the quality of life of children diagnosed with achondroplasia, the quality of life of parents of children with achondroplasia as well as correlations and determinants of children’s quality of life from both perspectives – child-reported quality of life and parent-reported quality of life. Both the children with achondroplasia and their parents rated the children’s quality of life significantly lower compared to a healthy reference population. We did not find any significant differences in the emotional domain in the child-report. The emotional domain includes expressions like feelings of anxiety, sadness, anger, worry, and sleep difficulties. In this domain, the children with achondroplasia reported quality of life comparable to a healthy reference population. A German-Swedish study is investigating the quality of life of children born with esophageal atresia using the PedsQL 4.0™ even reported a significantly higher quality of life score on the emotional scale compared to a healthy reference population [39]. Patients with chronic illness showed improved coping with everyday stressors compared to the healthy controls [40]. Similarly, Rohenkohl, Bullinger [8] showed that children with achondroplasia reported the highest quality of life scores in the emotional domain. The affected children may have learned to accept themselves as they are and to be satisfied with themselves - even if they experience significant limitations in the domain of physical quality of life, as well as in the domains of school and social life. While these capacities and environments cannot be influenced by the children, the intrapersonal emotional attitude towards oneself can be modulated and strengthened by suitable coping strategies and social support from parents and friends.

Results of our previous study, shows that participants of interventions, which are based on the specific needs of the target population, resulted in a significant increase in quality of life in comparison to non-participating patients [12, 34]. Our current sample consists of members of an active patient organization, which offers frequent regional and national membership meetings as well as intensive support through employees and volunteers. This support system might have an impact on our results – especially on the copings strategies used by affected children as well as their parents and their social and emotional quality of life.

The reduced quality of life of children with achondroplasia can be explained by the physical restrictions and limitations as well as various challenges in daily life reported by children, adolescents and young adults with achondroplasia in focus group discussions and was confirmed by their parents’ perception [21]. Despite the physical demands that result from this for the parents, the parents of our sample showed no restrictions compared to a representative comparison group about their physical quality of life. Similarly, parents of children born with esophageal atresia reported no physical quality of life restriction compared with a reference population [29]. In particular, limitations of motor skills as well as practical life skills contributed significantly to an increased parental burden [41, 42]. In addition to the above mentioned physical strains, parents of children with chronic health conditions are also exposed to other stressors, such as stigmatization, feelings of guilt, and fears of the future, which can have a negative impact on the mental health of the parents [11]. The present sample of parents of children with achondroplasia highlighted this burden with a significantly reduced mental quality of life compared to a German reference group. These findings confirm the results of a meta-analysis from Teubert and Pinquart [18], in which they described the burden and impairments of parents of chronically ill children compared to parents of healthy children. Also, parents of children with rare health conditions reported this significantly reduced mental quality of life [29]. While the parents of the children and adolescents with achondroplasia in this sample reported reduced mental quality of life, parents of short statured children and adolescents diagnosed with endocrine short stature showed no restrictions of their quality of life compared to a healthy reference population. However, in the study, no differentiation was made between physical and mental quality of life [33]. We assume that the form of short stature (e.g. growth hormone deficiency and small for gestational age versus achondroplasia) plays an important role not on the children’s quality of life [12] but also on the perception of parental quality of life. Children with endocrine short stature are much less likely to be stigmatized than children with achondroplasia. Furthermore children with achondroplasia often suffer from many complications and concomitant limitations in everyday life. Accordingly parents have to afford more care for children with achondroplasia than for children with endocrine short stature.

The correlations between parent-reported children’s quality of life and parental quality of life showed significant results for both parental physical quality of life and parental mental quality of life. The higher the parental quality of life, the higher the parent-reported children’s quality of life. This also shows the correlation between parental physical quality of life and child-reported children’s quality of life. However, we found no significant association for parental mental quality of life and child-reported quality of life. [29] showed similar findings and reported significant correlations between parental quality of life and parent-reported children’s quality of life for families with children born with esophageal atresia.

Sociodemographic and clinical characteristics of the children showed no effect on the children’s quality of life for neither the child-report nor the parent-report. However, parental physical and mental quality of life predicted the parent-reported children’s quality of life significantly. This stresses the importance of the parental perception of their children’s quality of life, depending on the parental physical and mental constitution.

The influence of the parental quality of life on the parental assessment of the children’s quality of life should be investigated in future research to identify relevant factors that affect the parental ability to assess their child’s quality of life. The different roles in caregiving and stressors of mothers and fathers should be considered when investigating the caregiving burden of parents whose children are diagnosed with a rare disorder such as achondroplasia. An increased understanding of the specific situation of parents of a chronically ill child will help to obtain insight starting points for interventions, to provide tailored support for these parents. As parents are able to improve their adjustment to the illness of their child and to increase their own quality of life, their children’s well-being and health-related outcomes may improve [43].

For clinicians, it is therefore important to assess children’s quality of life from all possible perspectives - from the child as well as both parents – together with the parental quality of life to obtain a comprehensive view of the situation and to include all reports in decision-making processes.

Limitations to this study include the recruitment of a highly selective sample (drawn only from a national patient organization), the lack of data about non-participants, and the lack of data pertaining to the sociodemographic background of the families. Clinical data were also reported by parents only. In addition to these weaknesses, more mothers participated in this study than fathers. We enrolled only one parent per child in this study. For future research, it would be useful to include both parents to comprehensively represent the family situation and to be able to reveal possible differences in parental perception. Rare disorders, such as achondroplasia, are chronically debilitating. Thus special efforts are needed to address patients’ and parent’s needs. The results of this paper confirm a reduced quality of life in affected children. We would recommend that such quality of life assessment should not only include self- and parent-reported data as complementary sources of information; they should also consider the family context [44].

Our results reveal that the parents of this study sample showed a reduced mental quality of life - regardless of the height of their child. Thus, it is important to recognize that pediatric short stature affects not only the child but the family as a whole [45].

The diagnosis of achondroplasia and its consequences may influence the daily life of the entire family because they have to adapt to the child’s special needs. A straightforward implication of our study is the need to assess the child’s as well as the parental quality of life to develop family-centered psychosocial interventions, thus improving both children’s and parents’ adaptation outcomes.

## Conclusions

Achondroplasia is chronically debilitating. Thus special efforts are needed to address patients’ and parent’s quality of life needs. The chronic health condition may influence the daily life of the entire family because they have to adapt to the child’s special needs. Although young patients report higher quality of life in the self-report their parents, the comparisons with healthy reference groups show reduced quality of life both from the perspective of the parents and the perspective of the affected children and adolescents. The reduced mental quality of life of parents refers to the burden for the parents that can result from the child’s chronic health condition. Since parental quality of life has a significant impact on the parent-reported quality of life of the children, clinicians should not only focus on the child’s quality of life but also those of the parents. Therefore, it is crucial to consider the psychosocial situation of the whole family.

## Data Availability

The datasets used and/or analysed during the current study are available from the corresponding author on reasonable request.
